# Correspondence

**Published:** 1871-02

**Authors:** 


### CORRESPONDENCE.

——————————Feb. 5th, 1870.

*Messrs. Editors:* I have received the first number of the Medical Companion. I felt delighted, when I took it from the office. After a careful examination, I am pleased and feel that I owe you many, many thanks. I am profoundly impressed that it should be liberally sustained by the profession—sustained, because it fills a palpable void in the wants of the practitioner—a void which, through the Companion as a medium for every practitioner—a medium through which all good physicians can, by a little harmonious effort and co-operation, advance the interest and elevate the honor of the fraternity. My battle in life is certainly well nigh over. But I delight to contemplate the progress made in my day—to meet now and then with those vigorous and active in the profession—especially with those who look to and talk of new ideas, and new achievements in the science and in the practice, and it is the pleasures of such interviews, just enjoyed with some of my younger medical brethren that prompts me to indite you a few random thoughts.

Looking out on the world and the profession from my retreat, there are one or two sad features in the situation of the case, which are as discouraging as they seem hopeless. The great Healing Art is a lie unto itself, as long as the world—the people—are permitted or taught to believe that there are a half dozen or more separate and distinct systems of the practice of medicine, any one or all of which are correct. As long as Homœpathy, Eclecticism, Thompsonianism and Hydropathy, and laying on of hands all have sway, there must be quackery, imposition, fraud and popular and professional demoralization. There must be danger to life and health. There must be a large number of ignorant pretenders, with diplomas, and without diplomas—who live by deception, as to what they administer—as to what they can cure—who live by insinuating themselves by deception, by tales of marvellous cures, into even intelligent families—who, by disregarding all rules of propriety, which govern intercourse between professional brethren, undermine even honest and able physicians, and it has come to this, that local and itinerant quacks grow rich on the people's misfortunes, while the true, devoted and scientific Physician hardly lives.

To arrest these evils seems almost a hopeless case. I lament the situation of things, and will not keep silent. At whose door first lies this state of things? I must first complain of our ablest and greatest and best Physicians. Through years of devotion to the real science and the real duties of practice, they have, in the evening of their lives, found themselves, with high reputation, and if not in the enjoyment of wealth and luxury, surrounded by comfort and plenty. My mind can call up the names of such in every section of the State. What is their offence, what their fault of which I complain? It is, that they forget to look out and see how their noble calling—noble in their hands—is degraded by the characters and the acts to which I have alluded. They fail to give the weight of their presence, and the weight of their names and opinions to the truth—to Society and organization, in battling against these lies, which Medicine, under false names, is made to tell upon itself. They are not heard on the great questions in which the vital, Ethical, moral power of the Profession is assailed. They are not found talking and writing against these evils. They are not doing their duty. The young and active Physician, and the people, look to them as the authorized and legitimate teachers on the great issues of the day, in medicine. They expect them to lay down the principles which should govern elevated intercourse, not only between physician and physician, but between physician and people ; and for them to fail to speak and teach, is almost as reprehensible as a Pastor would be who neglects his church and his flock.

But enough for the present, Messrs. Editors. If you will tolerate these babblings of an old man, there are one or two other thoughts on this subject that I may give in another communication. I want to show the importance of right organization—more important now for the profession, than ever before—but never, now or heretofore—anything like it should be. I want to show how it can elevate the character of the individual Physician—how it can raise the character of the profession at large—how it can—yes, how, right organization and co-operation under it, can actually be made a mercy and protection to the afflicted and the ignorant—how, indeed, everything good in the present situation, and all that is attainable in the future, depends upon right organization—organization that is sustained—clung to—abided by—organization that will read out and condemn those who are faithless to it, and sustain those who are right, without fear, favor or affection.

DRS. POWELL & GOLDSMITH :—*Gentlemen :* The first number of your "MEDICAL COMPANION AND MONTHLY ADVISER," has just come into my possession. It is not my purpose to allude to the typographical execution of the work—that speaks for itself; neither do I deem it necessary to compliment the editors, so much as to congratulate the medical profession in the fact, that we have still in our fraternity men whose high sense of honor and regard for those principles which have characterized our Profession in the past, who are willing to make sacrifices of time and money, that our science may be sustained against the attacks of those who are but fungus growths, and of spurious origin. However mortifying may be the thought to the high-toned, scientific professional gentleman, it is nevertheless true, the science of Medicine, based upon principles as enduring as the "eternal hills," and honored by men of all past ages, has become, by many, in many places, a matter of "barter and trade," and those of whom we ought to expect better things, not unfrequently are found the most successful and untiring adepts in securing a lucrative practice, while others in the profession, who regard as sacred the obligations of *medical ethics*, would scorn to resort to such subterfuges. The question is, how shall these evils be removed? I answer, by the circulation of well-conducted Medical Journals among those who are unwilling to sacrifice the honor of their profession for pecuniary advantages, and a united effort in medical societies by a strict conformity of their membership to *medical ethics*, and a fraternal feeling for those alone of our brethren who place themselves upon these time-honored principles.

That there are many evils which cannot be successfully met through Medical Journals, all must admit. The impositions which are daily palmed off upon the people through the agency of quack doctors and their multiplied nostrums, have become alarming in their tendency, and destructive in their results. To arrest these evils and save the people, well-written essays upon these subjects should be published, and placed in the hands of every man and woman in the country, that they may be aroused to a sense of the dangers which surround them.

Gentlemen, I regard your "COMPANION," under your auspices and known abilities and energy—already a success—filled with editorials, concise, practical and plain, with selections from home and foreign journals, containing only the gems of thought, together with the experience of valuable correspondents from every section of our country, as an omen which promises much to the Profession, in the elevatian of our science, and the successful overthrow of empiricism. MEDICUS.

